# Association between previous inguinal hernia surgery and the risk of anastomotic leakage after colorectal surgery: nationwide registry-based study

**DOI:** 10.1093/bjsopen/zrad076

**Published:** 2023-08-31

**Authors:** Erik Axman, Henrik Holmberg, Martin Rutegård, Hanna de la Croix

**Affiliations:** The Queen Silvia Children´s Hospital, Department of Pediatric Surgery, Gothenburg, Sweden; Department of Surgery, Institute of Clinical Sciences, Sahlgrenska Academy, University of Gothenburg, Gothenburg, Sweden; Department of Epidemiology and Global Health, Umeå University, Umeå, Sweden; Department of Surgical and Perioperative Sciences, Surgery, Umeå University, Umeå, Sweden; Wallenberg Centre for Molecular Medicine, Umeå University, Umeå, Sweden; Department of Surgery, Institute of Clinical Sciences, Sahlgrenska Academy, University of Gothenburg, Gothenburg, Sweden; Sahlgrenska University Hospital/Östra Hospital, Department of Surgery, Gothenburg, Sweden

## Introduction

Colorectal anastomotic leakage is a frequent and dreaded complication after colorectal surgery. The reported rates of anastomotic leakage vary with anatomical location and ranges from 3 per cent for the right colon, 4–7 per cent for the remainder of the colon and 11–18 per cent for the rectum^1,2^. Anastomotic leakage increases the risk of mortality and cancer recurrence, as well as imposing substantial healthcare costs^3,4^.

The cause of anastomotic leakage is multifactorial, with a multitude of suggested risk factors, often varying between studies. Established preoperative risk factors include male gender, a low rectal anastomosis, absence of prophylactic antibiotics and preoperative radiotherapy^5^.

Anastomotic leakage is also associated with poor wound healing, which has also been reported in patients with hernias. Recent studies show that zinc-dependent matrix metalloproteinases (MMPs) such as MMP-9 are elevated in patients with anastomotic leakage^6,7^ as well as in patients with inguinal hernia^8,9^. Male gender is a risk factor for both inguinal hernia and anastomotic leakage^5^. Connective tissue diseases such as Ehlers–Danlos syndrome increase the risk of inguinal hernia development and spontaneous colonic perforations in addition to high rates of anastomotic leakage^10,11^. Patients with Ehlers–Danlos syndrome have mutations in the genes coding for type I, III and V collagen leading to imbalance in the extracellular matrix and poor wound healing. Hypothetically, patients with a previous inguinal hernia repair might have a connective tissue imbalance leading to increased risk of anastomotic leakage.

The aim of the study is to investigate whether a previous inguinal hernia operation increases the risk of anastomotic leakage after surgery for colorectal cancer using high-quality registry data.

## Methods

### Study design and data source

This observational cohort study combines data from two quality registers with national coverage in Sweden, the Swedish Hernia Register (SHR) and the Swedish Colorectal Cancer Registry (SCRCR).

The SCRCR has registered patients with rectal cancer since 1995 and colon cancer since 2007 and includes 99 per cent of all patients with colorectal cancer in Sweden^12^. Data registration is collected prospectively and contains information regarding the characteristics of the patient, any operation and postoperative follow up^13^. Postoperative complications within 30 days, or in hospital, of the primary operation are recorded and graded according to the Clavien–Dindo classification. Anastomotic leakage was the primary study outcome.

The SHR was founded in 1992 and is a non-mandatory quality register with a cover rate of approximately 97 per cent^14^. Information regarding patient characteristics, the surgical method used and postoperative complications is included in the register.

All registered patients operated because of colorectal malignancy with an anastomosis between 1 January 2007 and 31 December 2018 were included in the study. With the use of the personal identification number^15^, the colorectal cancer registry was linked to the SHR. Patients with prior inguinal hernia surgery registered in the SHR were defined as exposed, while patients with no previous inguinal hernia surgery were defined as unexposed. Extreme values, such as weight < 30 kg or >200 kg, or a length < 130 cm or >220 cm, were coded as missing.

### Statistical methods

The study hypothesis was that a hernia influences the risk of anastomotic leakage and hence a directed acyclic graph was chosen for selection of covariables to include in the logistic regression analysis (*Supplementary material*). These variables thus constituted a minimally sufficient adjustment set in order to evaluate the total effect.

A binary logistic regression was used with anastomotic leakage as the outcome and previous inguinal hernia repair as the exposure. Odds ratios (ORs) with 95 per cent confidence intervals (c.i.) were estimated. The model was adjusted for age (centred), time period for colorectal cancer surgery (divided into tertiles), ASA grade (I, II or III–IV), sex and BMI as cubic splines with three knots. To address missing data, we used multiple imputation using predictive mean matching on all variables used in the model. The regression analysis was performed on pooled data from 10 imputed data sets. All analyses were also performed on a complete cases data set regarding the variables included in the model.

Subgroup analyses comparing single and multiple hernia operations, and the effect of a medial hernia, rectal and colonic anastomosis, were performed and are available in *Supplementary material*.

Statistical analyses were done using R Statistical Software (v4.1.2; R Core Team 2021) and MICE (Multivariate Imputation by Chained Equations in R) was used to perform multiple imputations.

## Results

A total of 42 762 patients who underwent colorectal cancer surgery with an anastomosis between 1 January 2007 and 31 December 2018 were identified in the SCRCR. In the SHR, 2041 of these patients were registered as having had an inguinal hernia repair before colorectal surgery. Basic characteristics of the included individuals are presented in *Table 1*. The mean age of all patients was 72 years, and most patients had a stage II or stage III cancer at the time of colorectal cancer surgery. Patients with and without previous inguinal hernia repair were similar regarding age, BMI, ASA grade, tumour location, preoperative radiotherapy, preoperative chemotherapy and clinical tumour stage.

**Table 1:** Baseline characteristics in patients undergoing colorectal cancer surgery between 2007 and 2018 in Sweden

	Patients without previous inguinal hernia surgery (*n* = 40 721)	Patients with previous inguinal hernia surgery (*n* = 2041)	Overall (*n* = 42 762)
**Sex**			
Male	19 630 (48.2%)	1888 (92.5%)	21 518 (50.3%)
Female	21 091 (51.8%)	153 (7.5%)	21 244 (49.7%)
**Age (years), median (i.q.r.)**	72.0 (64–79)	75.0 (69–81)	72.0 (64–79)
Missing	10 (0%)	0 (0%)	10 (0%)
**BMI, median (i.q.r.)**	25.4 (19.8–31.0)	24.7 (20.4–29.1)	25.4 (19.8–31.0)
Missing	3987 (9.8%)	172 (8.4%)	4159 (9.7%)
**ASA**			
I	6329 (15.5%)	226 (11.1%)	6555 (15.3%)
II	21 607 (53.1%)	1085 (53.2%)	22 692 (53.1%)
III–IV	11 888 (29.2%)	692 (33.9%)	12 580 (29.4%)
Missing	897 (2.2%)	38 (1.9%)	935 (2.2%)
**Tumour location**			
Colon	33 186 (81.5%)	1653 (81.0%)	34 839 (81.5%)
Rectum	7519 (18.5%)	388 (19.0%)	7907 (18.5%)
Missing	16 (0%)	0 (0%)	16 (0%)
**Preoperative radiotherapy**	4482 (11.0%)	229 (11.2%)	4711 (11.0%)
Missing	111 (0.3%)	3 (0.1%)	114 (0.3%)
**Preoperative chemotherapy**	2034 (5.0%)	94 (4.6%)	2128 (5.0%)
Missing	94 (0.2%)	3 (0.1%)	97 (0.2%)
**Cancer stage**			
Stage I	7019 (17.2%)	364 (17.8%)	7383 (17.3%)
Stage II	13 895 (34.1%)	723 (35.4%)	14 618 (34.2%)
Stage III	13 346 (32.8%)	665 (32.6%)	14 011 (32.8%)
Stage IV	4031 (9.9%)	170 (8.3%)	4201 (9.8%)
Missing	2430 (6.0%)	119 (5.8%)	2549 (6.0%)
Unilateral inguinal hernia	–	1709 (84%)	–
Bilateral inguinal hernia	–	332 (16%)	–
Lateral inguinal hernia	–	1116 (55%)	–
Medial inguinal hernia	–	679 (33%)	–
Combined medial and lateral inguinal hernia	–	180 (9%)	–
Other	–	66 (3%)	–
Single operation for inguinal hernia	–	1538 (75%)	–
Multiple operations for inguinal hernia	–	503 (25%)	–
Age at time of inguinal hernia surgery (years), median (i.q.r.)	–	67.8 (61.0–74.5)	–
Time between inguinal hernia and colorectal cancer surgery (years), median (i.q.r.)	–	7 (3–11)	–

i.q.r., interquartile range.

During the study period, a total of 1998 patients (4.7 per cent) had postoperative anastomotic leakage. Leakage rate was comparable in patients with and without prior inguinal hernia surgery, 4.8 per cent (97/2041) and 4.7 per cent (1901/40 721) with no significant difference (*Table 2*). Patients with or without previous inguinal hernia repair had similar postoperative outcomes regarding postoperative bleeding, postoperative infection, reoperation within 30 days due to any cause and the need for postoperative care in the intensive care unit (*Table 2*).

**Table 2:** Postoperative outcomes in patients undergoing colorectal cancer surgery between 2007 and 2018 in Sweden

	Patients without previous inguinal hernia surgery (*n* = 40 721)	Patients with previous inguinal hernia surgery (*n* = 2041)	Overall (*n* = 42 762)	Odds ratio or mean difference* (95% c.i.)	*P*
Anastomotic leakage	1901 (4.7%)	97 (4.8%)	1998 (4.7%)	1.02 (0.83–1.26)	0.860
**Perioperative bleeding (ml), median (i.q.r.)**	150 (50–300)	150 (50–300)	150 (50–300)	11.38* (−9.45–32.22)	0.284
Missing	1792 (4.4%)	102 (5.0%)	1894 (4.4%)		
Postoperative infection	2358 (5.8%)	133 (6.5%)	2491 (5.8%)	1.14 (0.95–1.36)	0.170
**Reoperation within 30 days**	3427 (8.4%)	193 (9.5%)	3620 (8.5%)	1.14 (0.98–1.33)	0.097
Missing	145 (0.4%)	9.00 (0.4%)	154 (0.4%)		
**Postoperative care at the intensive care unit**	2422 (5.9%)	133 (6.5%)	2555 (6.0%)	1.10 (0.92–1.32)	0.296
Missing	168 (0.4%)	6.00 (0.3%)	174 (0.4%)		

i.q.r., interquartile range. Odds ratio calculated with unadjusted logistic regression analysis. Difference in perioperative bleeding calculated with independent samples *t*-test.

There was no increased risk for anastomotic leakage after inguinal hernia repair with an adjusted OR of 0.90 (95 per cent c.i.: 0.73–1.12), *P* = 0.356 in logistic regression analysis. A complete cases analysis rendered similar results, with an adjusted OR of 0.88 (95 per cent c.i.: 0.70–1.10), *P* = 0.265.

The number of previous inguinal hernia operations, the site of colorectal resection (colon *versus* rectum) or the presence of a medial inguinal hernia did not alter the results (*Supplementary material*).

## Discussion

In this large cohort study combining data from two national quality registers in Sweden, the authors found no association between previous inguinal hernia repair and anastomotic leakage after colorectal cancer surgery. To the authors' knowledge no previous reports have been published assessing the association between inguinal hernia and the risk of anastomotic leakage.

Inguinal hernias and anastomotic leakage have been reported to be linked with alterations in the connective tissue metabolism. In the current study, there was no clear association between inguinal hernia and the risk of anastomotic leakage. The origin of inguinal hernias is multifactorial and it is probably only a subset of inguinal hernias that are caused by an abnormal collagen turnover. Medial inguinal hernias with an intact internal inguinal ring and a weakness in the abdominal wall could represent such a subgroup. For instance, medial inguinal hernia seems to be associated with colonic diverticulosis^16^. However, in our study no association was found between anastomotic leakage and medial hernias either.

There are also hypotheses regarding the association between inguinal hernia and abdominal aortic aneurysm^17^, haemorrhoids and varicose veins. However, no clear associations have been confirmed, and as all these conditions are frequent, it could be a matter of coincidence.

The frequency of anastomotic leakage was 5 per cent in the current study, which is low compared with other studies^1,2^. This can in part be explained by the fact that over 80 per cent of the patients had surgery for colon cancer, which is associated with a lower risk of anastomotic leakage compared to rectal cancer. Another possible explanation is the underreporting of anastomotic leakage in the SCRCR as was shown in previous studies^18,19^.

A deeper understanding regarding the pathogenesis of inguinal hernia and the diseases associated with it will undoubtedly give the surgeon better opportunity to tailor the treatment for the individual patient.

## Supplementary Material

zrad076_Supplementary_Data

## Data Availability

The data that support the findings of this study are collected from the Swedish Hernia Register and the Swedish Colorectal Cancer Register. Data are therefore not publicly available and cannot be requested from the authors. To access the data a separate application must be made to each register. This original article is not based on previous communication at any conference or meeting.
