# Supplementary material for: Association between previous inguinal hernia surgery and the risk of anastomotic leakage after colorectal surgery: nationwide registry-based study
Source: BJS Open. 2023 Aug 31;7(4):zrad076. doi: 10.1093/bjsopen/zrad076 (PMC10469297; doi:10.1093/bjsopen/zrad076)
Supplement: zrad076_Supplementary_Data [file zrad076_supplementary_data.docx]

**Previous inguinal hernia surgery does not increase the risk of anastomotic leakage after colorectal surgery**

Erik Axman^1,2^, Henrik Holmberg^3^, Martin Rutegård^4,5^, Hanna de la Croix^2,6^

1 The Queen Silvia Children´s Hospital, Department of Pediatric Surgery, Gothenburg, Sweden

2 Department of Surgery, Institute of Clinical Sciences, Sahlgrenska Academy, University of Gothenburg, Sweden

3 Department of Epidemiology and Global Health, Umeå University, Umeå, Sweden

4 Department of Surgical and Perioperative Sciences, Surgery, Umeå University, Umeå, Sweden

5 Wallenberg Centre for Molecular Medicine, Umeå University, Umeå, Sweden

6 Sahlgrenska University Hospital/Östra Hospital, Department of Surgery, Gothenburg, Sweden

Correspondence and requests for reprints can be addressed to:

Erik Axman

The Queen Silvia Children´s Hospital, Department of Pediatric Surgery, Gothenburg, Sweden

Behandlingsvägen 7, 416 50, Gothenburg, Sweden

[erik.axman@vgregion.se](mailto:erik.axman@vgregion.se)

004631-3435141

**Supplementary Materials - Index**

| **Supplementary Methods** |  |
| --- | --- |
| Additional methods, subgroup analysis. | *page 2* |
| **Supplementary Results** |  |
| Additional results, subgroup analysis. | *page 3* |
| **Supplementary Figures and Tables** |  |
| Figure 1 |  |
|  | *page 4* |
|  |  |
|  |  |
|  |  |
|  |  |
|  |  |

**Supplementary Methods**

To address the possibility of a more pronounced effect in patients with multiple hernia operations we made a sensitivity analysis dividing the exposure in to two different groups. In this analysis we compared patients with one operation to patients with two or more operations for inguinal hernia. Again, using a binary logistic regression model adjusted for the same covariables as in the primary analysis.

In a second subgroup analysis, differences between a colonic and a rectal anastomosis were analyzed, separating the cohort depending on tumor site.

In a final subgroup analysis, we analyzed the difference between a direct inguinal hernia and no previous hernia repair on the risk of anastomotic leakage after colorectal cancer surgery.

**Supplementary Results**

In a sensitivity analysis aiming to investigate a potential dose-response relationship, patients with two or more inguinal hernia operations prior to colorectal surgery had an increased anastomotic leakage rate of 5.8%, compared to 4.7% in patients without hernia operations and 4.4% in patients with only one registered inguinal hernia repair. Here, the adjusted analyses showed, in comparison to no previous inguinal hernia surgery patients, an OR of 0.84 (95% CI: 0.65–1.08) p = 0.165 in patients with one previous operation and an OR of 1.11 (95% CI: 0.76–1.64) p = 0.568 in patients with two or more operations.

In individuals with a colonic and rectal anastomosis the rate of anastomotic leakage was 4% and 9%, respectively. There was no difference in the rate of anastomotic leakage with or without a previous hernia operation for any location. The binary logistic regression analysis with leakage as the outcome showed an adjusted OR of 0.87 (95% CI: 0.65–1.14) p = 0.331 and an adjusted OR of 0.89 (95% CI: 0.59–1.28) p = 0.537 for colonic and rectal anastomoses, respectively.

In individuals with a direct inguinal hernia, the rate of anastomotic leakage was 5.2% compared to individuals with no previous inguinal hernia surgery where 4.7% hade an anastomotic leakage. In the binary logistic regression analysis, the adjusted OR is 0.87 (95% CI: 0.42–1.80) p = 0.702.

**Supplementary Figures and Tables**

Figure 1. Directed acyclic graph showing selection of covariables.

(BMI=Body Mass Index), (ASA=The America Society of
Anesthesiologist), (TNM=TNM Classification of Malignant Tumours).
Color code: Blue=Outcome or ancestor of outcome, Green=Exposure or ancestor of exposure, Red= Factor influencing both
exposure and outcome, White= Other variable not influencing exposure or outcome.

**
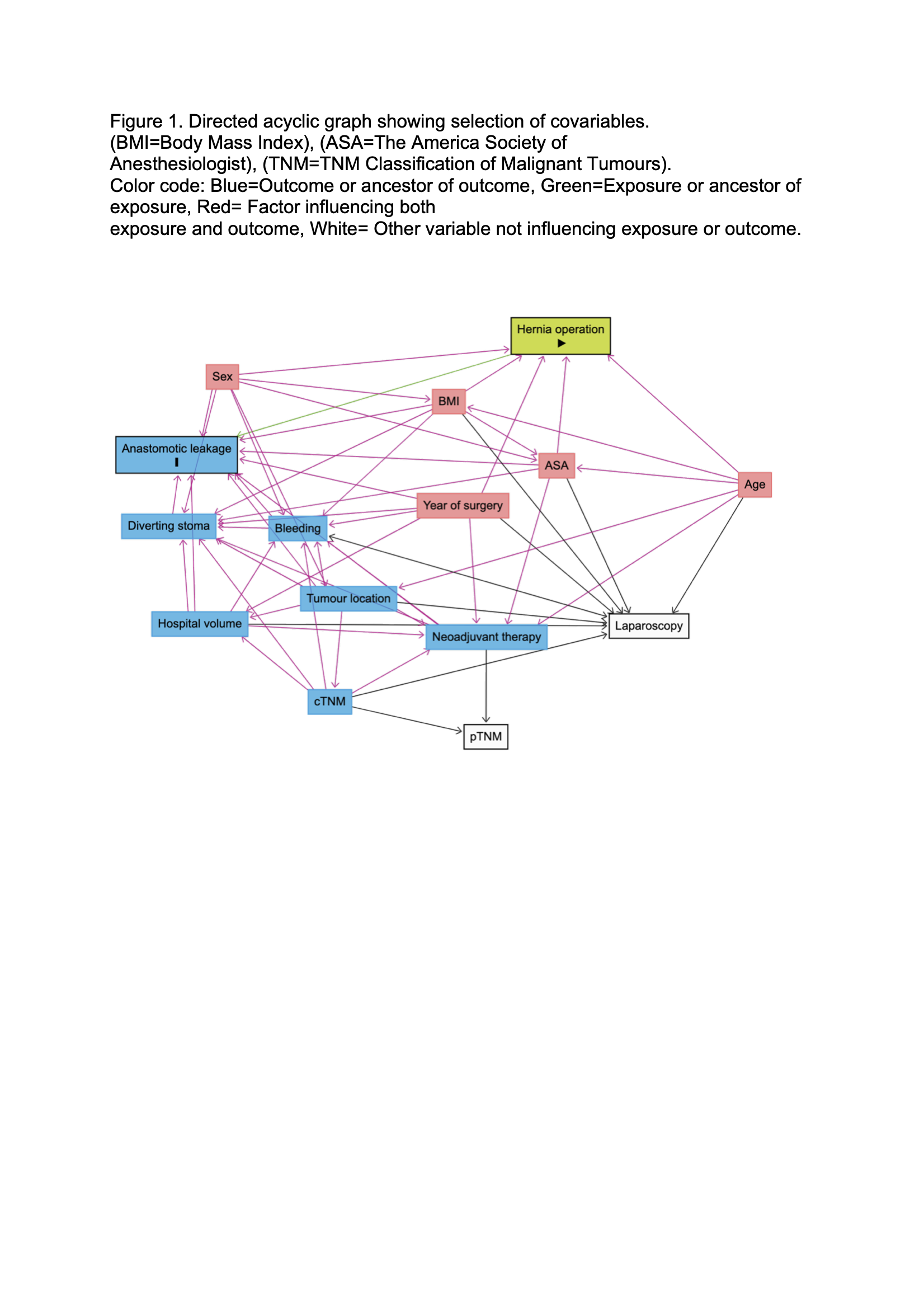
**
